# Rapid Anæthesia, by Ether

**Published:** 1885-07-20

**Authors:** 


**Rapid Anæsthesia by Ether.**—Dr. A. F. Muller, in the Med. News of April, 1885, describes a rapid method of anæsthesia free from mal-effects as follows:

The following method of rapid anæsthesia by ether was suggested to me seven or eight years ago by the thought that the great length of time often consumed in etherizing patients was due to the fact of the frequent interruptions neccessary to replenish the cone or towel used for the purpose, and the consequent partial recovery of the patient. To obviate the difficulty and obtain a continuous flow of pure ether vapor, I had made an apparatus, consisting of two halves of a rubber foot-ball, sewed together at the edges and connected by a tube with a bottle containing ether, which is plunged into a bucket of hot water. Ether boils at 98°, and vapor passes over steadily and rapidly, and is inhaled by the patient, whose face is covered by the inhaler, protected by a clean towel.

The result has been surprising, eighteen cases have been etherized by this method within the last three months at the Germantown Hospital. In none of the cases was there nausea previous to anæsthesia ; one at least came to the house the morning of the operation, having eaten a hearty breakfast. In most cases no struggling, and if so, only slight ; no stage of excitement. In cases that require only a few moments for operation the patient wakes up nearly as quickly as after a nitrous oxide. After the patient is etherized, the amount passing over can be regulated by a stopcock at the bottle end of the tube.

The apparatus I have used is very crude, made only for the purpose of experiment, and I am having an improved one made, which I hope will be more satisfactory in some of its details.

The quantity of ether used to produce complete insensibility in no case exceeded three ounces ; in some it was less than an ounce and a half.—*The Medical Age.*

